# Simulation-based surgical education for glaucoma versus conventional training alone: the GLAucoma Simulated Surgery (GLASS) trial. A multicentre, multicountry, randomised controlled, investigator-masked educational intervention efficacy trial in Kenya, South Africa, Tanzania, Uganda and Zimbabwe

**DOI:** 10.1136/bjophthalmol-2020-318049

**Published:** 2021-01-25

**Authors:** William H Dean, John Buchan, Stephen Gichuhi, Heiko Philippin, Simon Arunga, Agrippa Mukome, Fisseha Admassu, Karinya Lewis, William Makupa, Juliet Otiti, Min J Kim, David Macleod, Colin Cook, Matthew J Burton

**Affiliations:** 1 International Centre for Eye Health, Department of Clinical Research, London School of Hygiene and Tropical Medicine, London, UK; 2 Ophthalmology, University of Cape Town Faculty of Health Sciences, Observatory, Western Cape, South Africa; 3 Ophthalmology, University of Nairobi College of Health Sciences, Nairobi, Kenya; 4 Eye Centre, Faculty of Medicine, University of Freiburg, Freiburg im Breisgau, Baden-Württemberg, Germany; 5 Ophthalmology, Mbarara University of Science and Technology, Mbarara, Uganda; 6 University of Zimbabwe College of Health Sciences, Harare, Zimbabwe; 7 Department of Ophthalmology, University of Gondar, Gondar, Ethiopia; 8 Ophthalmology, Salisbury Hospital NHS Foundation Trust, Salisbury, Wiltshire, UK; 9 Ophthalmology, Kilimanjaro Christian Medical Centre, Moshi, Tanzania, United Republic of; 10 Ophthalmology, Makerere University Faculty of Medicine, Kampala, Uganda; 11 Tropical Epidemiology Group, Faculty of Infectious Disease Epidemiology, London School of Hygiene and Tropical Medicine, London, UK; 12 Moorfields Eye Hospital NHS Foundation Trust, London, UK

**Keywords:** glaucoma, treatment surgery, medical education

## Abstract

**Background/Aim:**

Glaucoma accounts for 8% of global blindness and surgery remains an important treatment. We aimed to determine the impact of adding simulation-based surgical education for glaucoma.

**Methods:**

We designed a randomised controlled, parallel-group trial. Those assessing outcomes were masked to group assignment. Fifty-one trainee ophthalmologists from six university training institutions in sub-Saharan Africa were enrolled by inclusion criteria of having performed no surgical trabeculectomies and were randomised. Those randomised to the control group received no placebo intervention, but received the training intervention after the initial 12-month follow-up period. The intervention was an intense simulation-based surgical training course over 1 week. The primary outcome measure was overall simulation surgical competency at 3 months.

**Results:**

Twenty-five were assigned to the intervention group and 26 to the control group, with 2 dropouts from the intervention group. Forty-nine were included in the final intention-to-treat analysis. Surgical competence at baseline was comparable between the arms. This increased to 30.4 (76.1%) and 9.8 (24.4%) for the intervention and the control group, respectively, 3 months after the training intervention for the intervention group, a difference of 20.6 points (95% CI 18.3 to 22.9, p<0.001). At 1 year, the mean surgical competency score of the intervention arm participants was 28.6 (71.5%), compared with 11.6 (29.0%) for the control (difference 17.0, 95% CI 14.8 to 19.4, p<0.001).

**Conclusion:**

These results support the pursuit of financial, advocacy and research investments to establish simulation surgery training units and courses including instruction, feedback, deliberate practice and reflection with outcome measurement to enable trainee glaucoma surgeons to engage in intense simulation training for glaucoma surgery.

**Trial registration number:**

PACTR201803002159198.

## Introduction

Globally, 36 million people are blind, and glaucoma is the third leading cause after cataract and uncorrected refractive error.^1^ Trabeculectomy remains a gold standard and cost-effective surgical management for glaucoma.^2 3^ Surgical treatment of glaucoma may be a first-line management strategy in moderate cases and is essential for treating advanced and severe glaucoma.^4 5^ Despite the need, there is a reticence among many ophthalmologists to perform trabeculectomy, most easily attributable to lack of surgical training in glaucoma procedures and challenges in patient safety performing delicate surgery on what may be a patient’s only seeing eye.^6–8^ The number of trabeculectomies being performed is reducing and this has a further impact on training.^9^ The use of glaucoma drainage devices has increased over the past three decades, and more recently minimally invasive glaucoma surgery (MIGS) has also played a role in the reduced number of trabeculectomies performed.^10^

An international survey of 38 countries showed a glaucoma surgical rate of 139 (range 3–500) surgeries performed per million population per year.^11^ There is a need to perform more glaucoma surgeries in order to reduce the burden of avoidable blindness. Despite this need, only half of final year trainees in the UK are confident in performing surgical trabeculectomy.^6^ The median number of glaucoma surgeries performed by senior trainee ophthalmologists (soon to become consultants) in sub-Saharan Africa was 1.^7^ Less than half of consultant ophthalmologists in Scotland and West Africa perform any glaucoma surgery.^12 13^ Hence, training of eye surgeons in glaucoma surgery, particularly trabeculectomy, needs to be increased while aiming for high-quality surgical education to ensure the best possible outcomes of a technically challenging operation.

Simulation offers an environment in which learners can train until they reach specified levels of competence.^14^ Simulation-based surgical education can rapidly increase surgical skills, decrease complication rates, provide a safe and relaxed environment to learn in, and enable sustained deliberate practice.^15^

**Figure 1:**
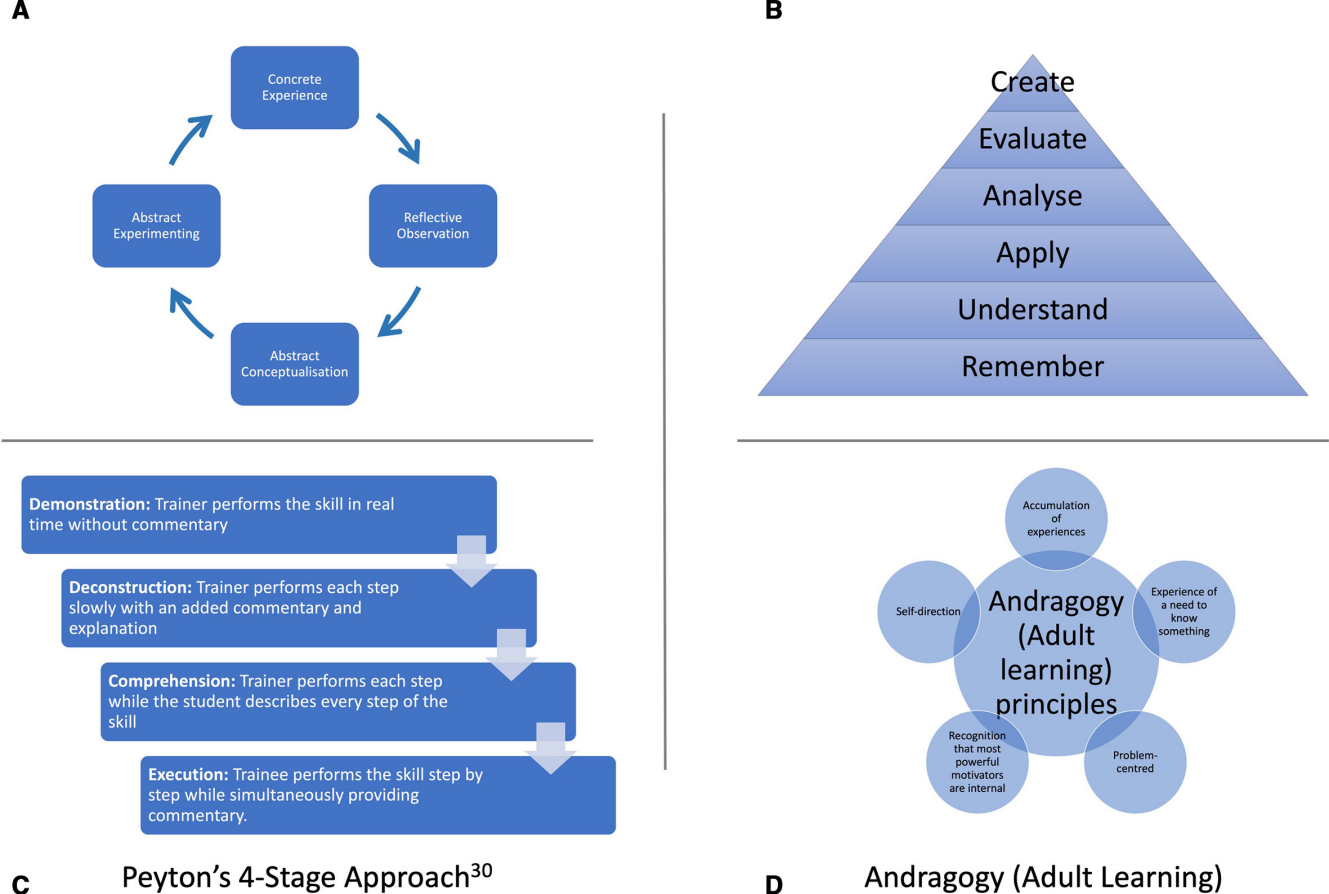
Educational frameworks: (A) Kolb’s learning cycle^16^; (B) Bloom’s taxonomy of learning^29^; (C) Peyton’s four-stage approach^30^; (D) andragogy (adult learning).

David Kolb^16^ developed the constructivist perspective of ‘experiential learning’ as a cycle of active experimentation, concrete experience, reflective observation and abstract conceptualisation (figure 1A). Reflection (or reflective observation) is a key aspect of experiential learning and can be included in simulation training courses. Ericsson^17 18^ highlighted the role of ‘deliberate practice’ being distinct from work or play, and that for expertise to be attained this practice should be deliberate, sustained (over years) and characterised by the desire to improve. This sustained deliberate practice is also a key facet in a simulation training intervention, although aimed towards the stage of ‘competence’ rather than ‘expertise’ in the Dreyfus model of skills acquisition.^19^

Numerous simulation models have been used in ophthalmic surgical education, predominantly for cataract.^20–23^ An apple peel and cellophane model has been used for trabeculectomy training with scleral flap construction.^24^ Artificial model eyes are available for trabeculectomy, drainage devices and MIGS.^25 26^ However, the impact of intensive simulation-based surgical education has not yet been comprehensively proven for ophthalmic surgical training and certainly not for glaucoma surgical training.^23^ We therefore designed and conducted the GLAucoma Simulated Surgery (GLASS) trial. The aim was to evaluate the effect of intense simulation-based surgical education in glaucoma surgery on surgical competence, confidence and live surgery outputs compared with conventional training alone.

## Methods

### Study design

We designed a randomised controlled, parallel-group efficacy trial. Participants were randomised to one of two arms, with intended 1:1 allocation ratio. The predefined primary outcome was the 3-month surgical competency score. There were no changes to the methods after trial commencement. The study protocol is available at https://researchonline.lshtm.ac.uk/id/eprint/4654987.

### Participants

We enrolled trainee ophthalmologists from six university postgraduate training institutions in Kenya, Tanzania, Uganda, South Africa and Zimbabwe, selected according to inclusion criteria of having performed no trabeculectomy procedure as primary surgeon and part-performed or assisted in less than five. Trainees were in their second, third or fourth year of training. Training was similar in each centre in terms of duration (3–4 years) and glaucoma surgical experience. Informed written consent was obtained. Trainees in both arms continued with their regular training during the study period. Control arm participants were offered no placebo intervention, but were offered the same educational intervention in Cape Town after the initial 1-year follow-up period. Training, travel and accommodation expenses were funded; however, participants were given no further incentives or compensation.

### Prerandomisation baseline assessment

Following enrolment, participants were assessed for baseline surgical competence. This involved performing three simulation trabeculectomy procedures on artificial eyes or parts thereof as far as known by the participant. The video recordings were anonymised and remotely assessed using the Ophthalmic Simulated Surgical Competency Assessment Rubric (Sim-OSSCAR).^27^ A knowledge test was administered comprising 30 multiple choice questions on glaucoma, further adding to baseline participant data.

### Randomisation

Each of the six university training centre recruitment sites had its own separate randomisation sequence. The randomisation sequences were computer-generated centrally by a statistician based at the London School of Hygiene and Tropical Medicine, who was independent of all other aspects of the trial. We randomly allocated candidates at the site level into batches of two or four trainees, with equal numbers of intervention and control allocations in each batch. Preprinted allocation cards which specified the centre, batch group, unique identifier and allocation (intervention or control) were concealed inside opaque sealed envelopes. This ensured that the principal investigator, coinvestigator and participants had no prior knowledge of the allocation until the envelopes were opened. All the envelopes in the batch had an identical external appearance and batch label code. All trainees in the batch were each invited to simultaneously select and open one of the envelopes and to reveal their allocation card. If an odd number of participants were identified in a centre, the final one was invited to select one of two identical envelopes in a batch of two. This ensured randomisation as all candidates had an equal chance of being in either arm.

### Intervention

The intervention course was based on adult educational theory, aiming where possible towards the higher cognitive functions of Bloom’s taxonomy of learning (figure 1B).^28 29^ The trabeculectomy procedure was deconstructed in short steps, which were taught using Peyton’s four-stage approach to teaching a practical skill.^30^ A weakness of Peyton’s four-stage approach is that it does not integrate theory with practice, and a modified three-step approach was used (commonly omitting step 3) combined with prior statement of objectives and clinical reasoning and immediate feedback (figure 1C). Feedback was given to participants while they engaged in sustained deliberate practice of a particular step.^17^ We used both low-cost, moderate-fidelity materials, including foam for meticulous suturing practice and apple peels for scleral flap construction.^24^ Once all parts of the surgical procedure were covered to a level of competence, the full procedure was performed on high-fidelity synthetic simulation surgery eyes (PS-OS-010, Phillips Studio, Bristol, UK),^25^ following a round of mental rehearsal.^31^ The procedures were performed using Zeiss Stemi 305 microscopes (Carl Zeiss Microscopy, Jena, Germany). The microscopes were equipped with cameras and linked to a central router and local area network. The Zeiss Labscope App (V.2.8.1) on iPads completed the digital classroom, allowing surgeons to record their performance. On completion of a simulated trabeculectomy, trainees engaged in reflective learning by watching the performance back on the iPad and grading against the Sim-OSSCAR.^27 32^ Key andragogy principles, including problem-centred (rather than topic-centred) learning, internal motivation and self-direction, were incorporated (figure 1D). A more detailed description of the intervention is available in the online supplemental appendix.

### Outcomes

Participants were followed up at 3 months postintervention, at 1 year and at 15 months. Outcomes were assessed from video recordings of the simulation surgical procedures. Each video was independently graded by two masked graders who were experts in glaucoma surgery and had undergone familiarisation training using the Sim-OSSCAR. Video recordings of procedures were allocated a random seven-digit number, being the only identifiable information available for grading. Thus, assessors were masked to the participant’s identity, allocation arm, training institution, as well as timing of surgical assessment.

The primary outcome measure was the mean score of three masked assessments of simulation surgical performance using the Sim-OSSCAR^27^ at 3 months. The total possible score was 40 points per assessment. If data were missing from one assessment, then the mean of two or the result of one assessment was used. Live surgical training opportunities for trabeculectomy are sparse^7^ and were not part of the intervention in the GLASS trial. We aimed to assess any effect of the intervention over a reasonable period of time, rather than merely the final day of an intense training course; hence, 3 months was chosen for the primary outcome measure.

Secondary outcome measures included surgical competence scores on the final day of the intervention training course, at 12 months and at 15 months (being 3 months after the control group had received their training intervention). Control group participants received exactly the same 1-week training intervention as the intervention group, after the 12-month assessment. The maintenance of surgical skills learnt in a simulation environment assessed over different time points has been reported as a valid methodology, predominantly in laparoscopic virtual reality and box trainer simulation surgical education research.^33^ The number of surgical procedures (live trabeculectomy) performed as primary surgeon, as well as assisting surgeon, was reported for 12 months. These were self-reported retrospectively in a summary report after 12 months. Outcomes were recorded in terms of complications and surgical success (defined as intraocular pressure (IOP) <21 mm Hg at last assessment with no further treatment).

There were no changes to trial outcomes after the trial commenced. Additional exploratory analysis included surgeon confidence scores (on a 10-point Likert scale, anchored at 1=‘not confident at all’ and 10=‘very confident’) recorded at baseline and at 3, 12 and 15 months.

### Statistical analysis

Based on pilot data we calculated a sample of 23 individuals in each arm would have 80% power and 95% confidence to detect a significant difference. We aimed to recruit 25 per arm to provide 2 extra participants as modest loss to follow-up. The baseline characteristics of participants were tabulated and the distributions of these variables by treatment arm were compared to assess for imbalance.

The trial had a prespecified data analysis plan. Intention-to-treat (ITT) analysis was used for all outcome measures. The primary outcome was analysed by Wilcoxon rank-sum and a linear regression model for Sim-OSSCAR at 3 months, with trial arm as the exposure, adjusted for surgical training centre and baseline mean Sim-OSSCAR score. Secondary outcome measures were analysed by linear regression, as per the approach used for the primary outcome.

The number of surgeries performed over 1 year was analysed using Poisson regression, with trial arm as the exposure of interest, adjusting for training centre. Confidence rating scores (assessed at baseline and at 3, 12 and 15 months) were analysed using Wilcoxon rank-sum test.

An alpha level of p<0.05 was considered statistically significant for the primary outcome. A kappa coefficient of ≥0.75 for inter-rater agreement was considered excellent.^34^

Data were initially entered into Microsoft Excel (V.15.31). Statistical analysis was performed using Stata V.15.1. A data monitoring and trial advisory committee oversaw the study.

### Prevention of bias

It is accepted that there will be variability in individual participants’ inherent or natural surgical aptitude. All efforts were made to standardise the training offered to the ‘intervention’ participants (as well as the ‘control’ participants after the 1-year period). The intense simulation course was held in the same standardised surgical training unit at the University of Cape Town. The training was conducted by WHD.

It is recognised that surgical education is complex and multifaceted. However, every effort was made to reduce ‘contamination’ bias. A number of standard risk-of-bias criteria are suggested for randomised controlled trials (RCTs) (or studies with a separate control group). These are summarised in the online supplemental appendix table.

## Results

**Figure 2:**
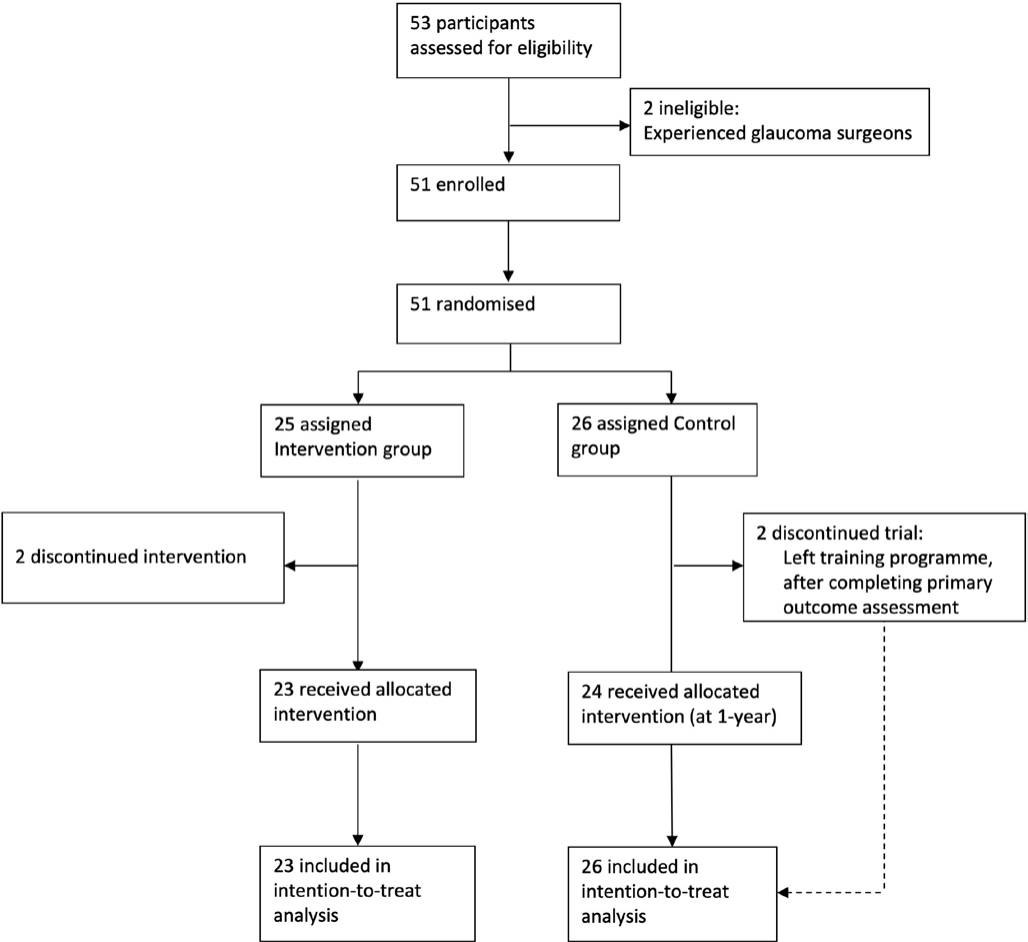
GLASS trial profile. GLASS, GLaucoma Simulated Surgery.

A total of 53 potential participants were assessed for eligibility. Fifty-one were recruited, with 25 allocated to the intervention group and 26 to the control group. Forty-nine were included in the final ITT analysis, with two dropouts from the intervention group. Figure 2 illustrates the trial profile. Two potential participants were excluded prerandomisation due to prior surgical experience. One intervention group participant failed to travel for the intervention training due to visa issues. Another participant completed only part of the intervention course and subsequently failed to respond to repeated invitations for follow-up.

Table 1 shows the demographic data of the participants. There was good balance between the two arms. All ophthalmology training programmes and countries contributed participants (Kenya 17, South Africa 2, Tanzania 12, Uganda 14 and Zimbabwe 4). There were no unintended effects in either arm.

A total of 604 videos were independently graded, of which 287 were directly included in the primary outcome measure analysis. Interobserver reliability correlation of outcome assessors showed a kappa correlation of video total scores of 0.83. The intraobserver agreement was 0.88.

**Table 1:** Baseline demographic characteristics of GLASS trial participants

Characteristics	All (N=49)	Intervention (n=23)	Control (n=26)	P value
Age, mean±SD	33.2±4.0	33.1±3.7	33.2±4.3	0.82
Sex, n (%)				
Female	23 (46.9)	12 (52.2)	12 (46.2)	0.67
Male	26 (53.1)	11 (47.8)	14 (53.8)	
Year of training, median (mean)	2 (2.5)	2 (2.5)	2 (2.4)	
MCQ score (%), mean±SD	75.7±9.7	77.4±8.5	74.2±10.6	0.29
Trabeculectomy procedures assisted or part-performed, mean (median)	0 (0)	0 (0)	0 (0)	

GLASS, GLAucoma Simulated Surgery; MCQ, multiple choice question.

The mean Sim-OSSCAR score at 3 months was 30.4 (76.1%, SD 4.4) and 9.8 (24.4%, SD 3.6) for the intervention and the control group, respectively. Those who received the training were estimated to have unadjusted scores of 20.6 points higher (95% CI 18.3 to 22.9) (p<0.001). The difference was 20.4 points higher (95% CI 18.7 to 22.2) with adjustment for baseline scores and training centre (p<0.001).

Surgical competency at 12 months was maintained by the intervention group: a mean score of 28.6 points (SD 3.9). The mean competency score of the control group was 11.6 (SD 4.4) (mean difference 17.0, 95% CI 14.8 to 19.4, p<0.001).

**Figure 3:**
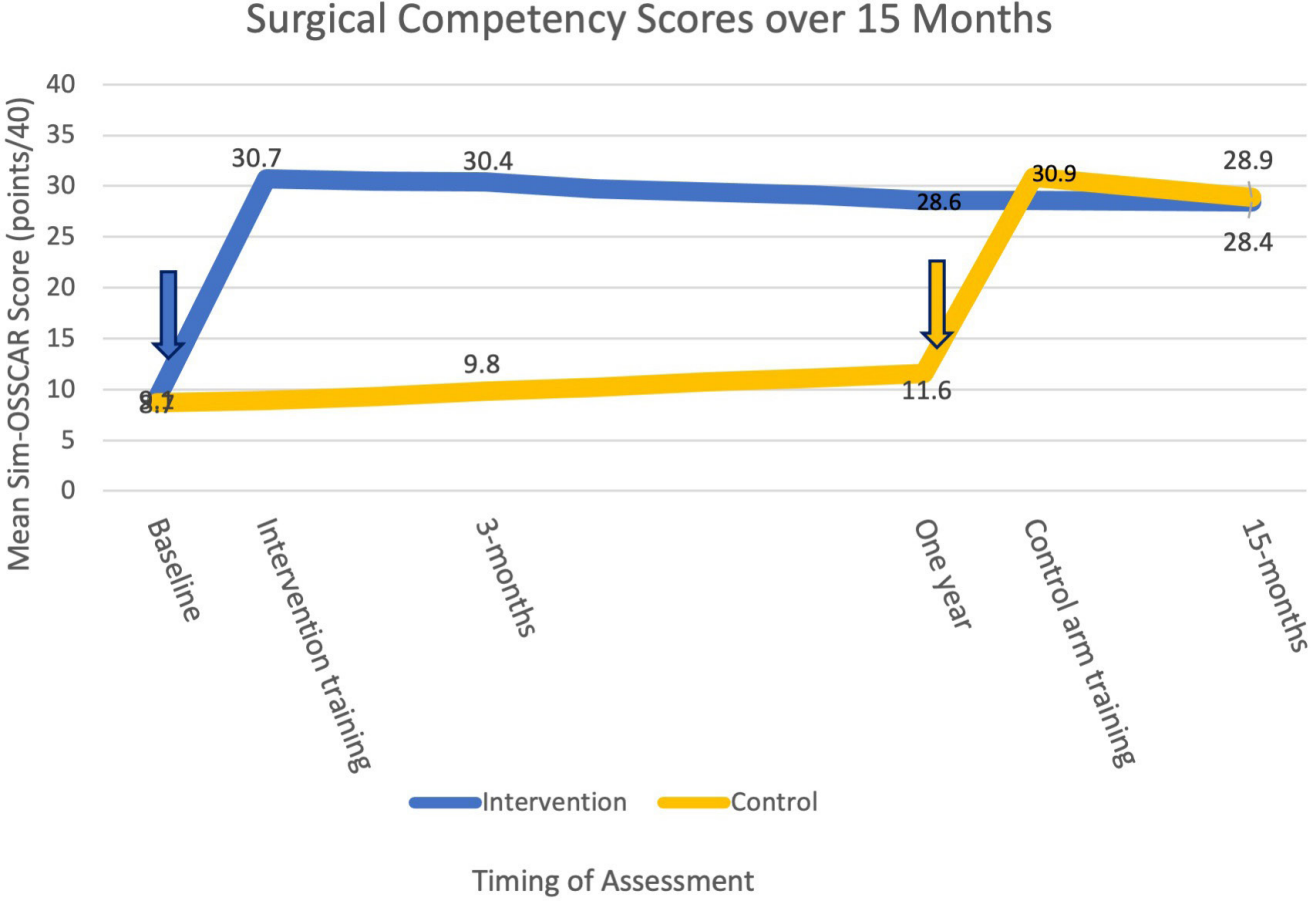
Surgical competency (score out of 40) over 15 months by arm. The arrows indicate the training course intervention. Sim-OSSCAR, Ophthalmic Simulation Surgical Competency Assessment Rubric.

Surgical competency was assessed on the final day of the training course for each group (figure 3). This increased from a baseline of 9.1 out of 40 (22.8%) to 30.7 (76.9%) (SD 5.1) for the intervention group. Before the control group undertook the training intervention (at the 12-month assessment), their mean competency score was 11.6 (29.0%) and this increased to 30.9 out of 40 (77.6%) (SD 3.7) at the end of the training course (p<0.001) (table 2, figure 3).

**Table 2:** Objective evaluation of Traceculectomy Sim-OSSCAR scores: intervention versus control groups

Timing of simulation trabeculectomy competency assessment	Intervention Score*, mean (%) (SD)	Control Score*, mean (%) (SD)	Difference† Score*	95% CI Score*	P value
Baseline	9.1 (22.6) (5.0)	8.7 (21.8) (3.7)	−0.3	−2.8 to 2.2	0.788
Final day of training course‡	30.7 (76.9) (5.1)		21.7	19.0 to 24.4	(0.117)‡
3-month (primary outcome)	30.4 (76.1) (4.4)	9.8 (24.4) (3.6)	20.6	18.3 to 22.9	<0.001
12-month	28.6 (71.5) (3.9)	11.6 (29.0) (4.4)	17.0	14.8 to 19.4	<0.001
Final day of training course‡		30.9 (77.6) (3.7)	19.2	17.3 to 21.0	(0.117)‡
15-month	28.4 (71.0) (2.1)	28.9 (72.3) (5.0)	−0.5	−7.1 to 6.0	0.873

*Score out of 40 points.

†Adjusting for training centre as a fixed effect.

‡Training course intervention was after 12 months for control participants (p=0.177 relates to intervention vs control on final day of training course).

Sim-OSSCAR, Ophthalmic Simulation Surgical Competency Assessment Rubric.

**Figure 4:**
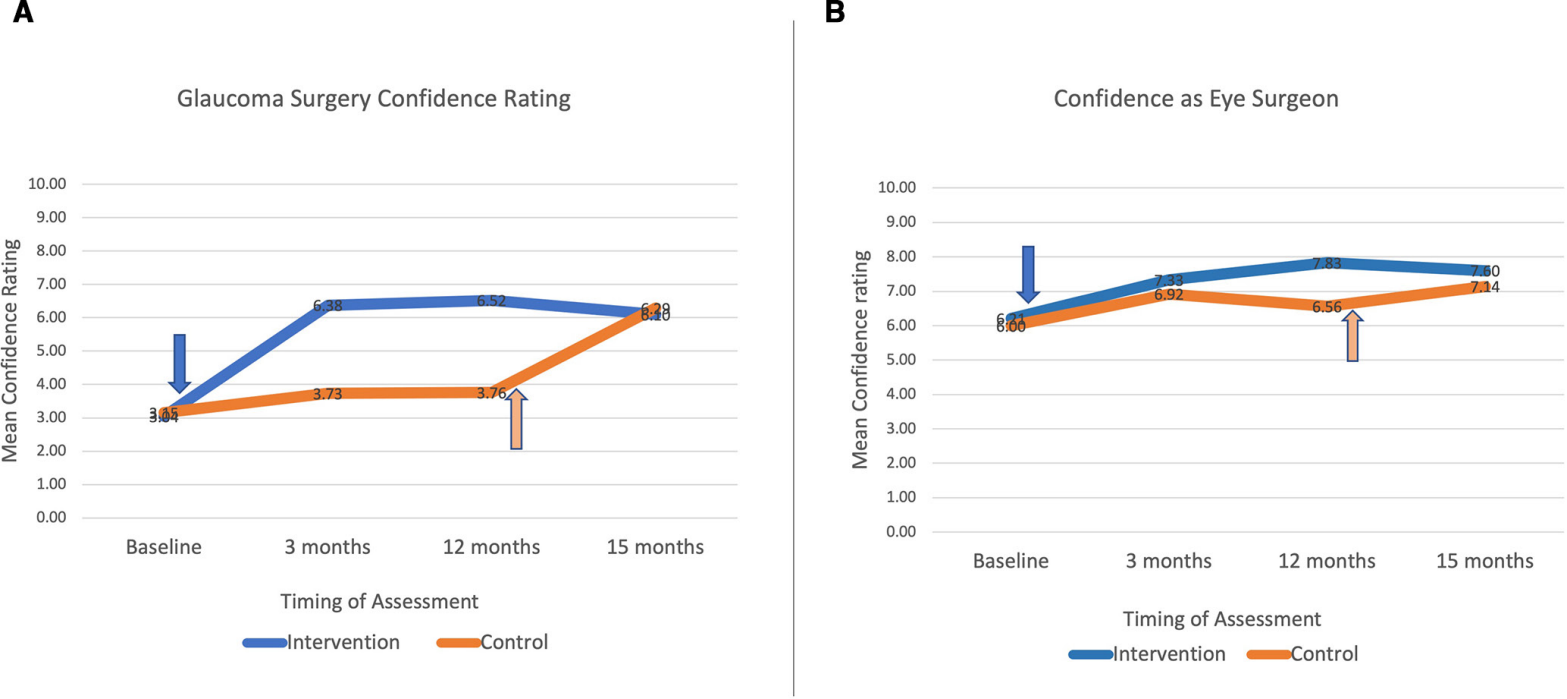
(A) Confidence rating in glaucoma surgery and (B) confidence rating as an eye surgeon.

The baseline mean self-reported confidence in ‘glaucoma surgical skills’ was 3.0 out of 10 for intervention and 3.2 for control participants (p=0.72). This increased to a mean of 6.4 and 3.7 at 3 months, respectively (p<0.001) (figure 4A). Confidence ‘as an eye surgeon’ was rated on the same 10-point scale. There was no difference at baseline or 3 months between the arms (p=0.38). At 12 months, the intervention group participants were more confident as eye surgeons: mean 7.86 vs 6.56 (p=0.022) (figure 4B).

The total number of trabeculectomies performed over 1 year was recorded for each participant. The intervention group trainees performed a mean of 3.2 live trabeculectomies as primary surgeon (median 2, range 0–15, IQR 0–4) in the year following the intervention training. In the same year period, control participants performed a mean of 0.15 (median 0, IQR 0–0). Poisson regression analysis, with trial arm as the exposure of interest, adjusting for training centre showed a large effect (p<0.001). Of the 26 control participants, 25 had performed zero trabeculectomy as primary surgeon, with only one having performed four supervised live surgeries. Of the 23 intervention participants, 14 (61%) had performed trabeculectomies (χ^2^ p<0.001). The incident ratio for the 1-year period showed intervention participants were 20.3 times more likely to perform surgery (p<0.001). The intervention trainees assisted in a mean of 4.8 trabeculectomies and the control group trainees assisted in a mean of 0.7 over the same 1-year period. Complications (including conjunctival leak and hypotony) were recorded for 12.2% (6 of 49) of the intervention group which performed surgeries. Surgical success (IOP <21 mm Hg, no further glaucoma treatment) was observed in 83.4% (41 of 49) of eyes. The number of surgeries performed by the control group (4 in total for all 26 participants) was too low for any meaningful comparative analysis.

## Discussion

The GLASS trial demonstrated that the intervention of an intense 5-day simulation-based training course successfully improved the main outcome of glaucoma surgical competence at 3 months. There is evidence from secondary outcomes that these benefits persisted over more than a year. There is further evidence that the quantity of live surgeries performed benefited from the intervention, as did self-reported confidence of participants in general and procedure-specific surgical ability.

It is likely that a combination of factors was related to the sustained increase in competence. After the training, participants were certainly more competent and confident in glaucoma surgery, but were also probably more motivated to perform supervised live surgery. Consultant ophthalmologists in collaborating training institutions would take notice of an increase in motivation and confidence and respond to a rapid and demonstrable increase in surgical competence of their trainees.

Ophthalmology training courses globally range from 3 to 7 years, and it is not possible from these data to determine the best timing of an intense simulation-based surgical educational intervention for glaucoma surgery. Competence, confidence and subsequent live surgical experience are linked, and therefore a recommendation for the best time of a GLASS training intervention could be the start of a glaucoma firm or rotation. Evidence from primary and secondary outcome measures of the GLASS trial indicates that the benefits of the training were very strong and equal for both the control and intervention group participants 1 year apart.

Limitations of the GLASS trial include the use of a simulation assessment of surgical competence. Both the Sim-OSSCAR for trabeculectomy and the live surgery ICO-OSCAR (International Council of Ophthalmology – Ophthalmology surgical competency assessment rubric) are validated competency assessment tools.^27 35^ However, it is perhaps also a strength that the ICO-OSCAR was not used as an outcome measure, as only one of the control participants performed any glaucoma surgery in the initial 1-year follow-up period. A strength of the GLASS trial is its RCT methodology, which to the authors knowledge is the first time ever applied to glaucoma simulated surgical education. Further strengths include standardised intervention training for all participants, and investigator masking and double assessment of all 604 simulation surgical videos.

Surgical education in glaucoma is challenging.^8 9 13 36^ Fewer glaucoma surgical procedures are being performed overall, the microsurgical procedure is intricate and requires meticulous technique, and long-term follow-up is needed beyond when a trainee would have moved on.^8 37^ Trainee ophthalmologists in Australia perform a mean of between 1.1 and 1.6 trabeculectomies per year,^8 38^ and trainees in the UK have a mean annual trabeculectomy rate of 0.5.^39^ Residents in the USA have completed a mean of 8.6 trabeculectomies by the end of their 3-year residency; however, two-thirds (67%) of residents begin operating as primary surgeon performing trabeculectomy only in their final year.^40^ The impact of curtailed hands-on glaucoma training opportunities is mitigated by the availability of subspecialty training fellowships in Australia, UK and USA.

Challenges in glaucoma management in sub-Saharan Africa include late presentation at an advanced stage of disease progression; lack of access to, affordability of, and adherence to medical therapy; low follow-up rates; and healthcare workforce shortages.^41^ It is imperative that general ophthalmologists be trained in glaucoma surgery and to a high standard considering the potential for surgical failure due to the propensity for scarring and the importance of good outcomes in a group of patients who may already be blind in the other eye. Many trainees will have finished their ophthalmology specialist training without having completed any glaucoma surgery and would then be less likely to perform many as a junior consultant. This would only act to keep the glaucoma surgical rate below the level needed to alleviate the burden of avoidable blindness due to advanced glaucoma.

Participants who received the training intervention in the GLASS trial went on to perform a greater number of live surgical trabeculectomy procedures in the year after the training intervention compared with control trainees. All participants benefited from a rapid and sustained increase in competence, thus making them more likely to maximise training opportunities when they arise.

Intense simulation training in glaucoma surgery affords a rapid and sustained increase in surgical competence and confidence as a surgeon, and impacts the number of live surgeries subsequently performed. It provides a calm environment in which to learn and practise the intricate and meticulous skills of surgical trabeculectomy. It provides a safe environment with no danger to patients. Surgical outcomes for trabeculectomy performed by intervention group participants were comparable with previous reports of resident-performed glaucoma surgery.^42^ However, rather than simply the availability of a simulator or artificial eyes as a simulation model, instruction, feedback, sustained deliberate practice and reflection with outcome measurement were all important aspects of the educational intervention. If used as a comprehensive educational package, simulation can play a pivotal role in training ophthalmic surgeons in advanced surgical techniques.

We now have the RCT-level evidence to suggest that it is an ethical, clinical and educational imperative for ophthalmology training institutions to pursue the use of intense simulation training in glaucoma to ensure trainees attain a benchmarked level of competence before operating on patients in a high-stakes, high-risk environment.

## Data Availability

Data are available upon reasonable request. For all reports (regardless of funding source) containing original data, WHD had full access to all the data in the study and takes responsibility for the integrity of the data and the accuracy of the data analysis. Participants were assured of confidentiality and anonymity of individual outcome assessments. Anonymised and de-identified data and statistical codes may be made available via the corresponding author.
